# Characterization of Pathogenesis and Inflammatory Responses to Experimental Parechovirus Encephalitis

**DOI:** 10.3389/fimmu.2021.753683

**Published:** 2021-11-25

**Authors:** Ming-Wei Jan, Hong-Lin Su, Tsung-Hsien Chang, Kuen-Jer Tsai

**Affiliations:** ^1^ Institute of Clinical Medicine, College of Medicine, National Cheng Kung University, Tainan, Taiwan; ^2^ Department of Medical Education and Research, Kaohsiung Veterans General Hospital, Kaohsiung, Taiwan; ^3^ Department of Life Sciences, Agriculture Biotechnology Center, National Chung-Hsing University, Taichung, Taiwan; ^4^ Department and Graduate Institute of Microbiology and Immunology, National Defense Medical Center, Taipei, Taiwan; ^5^ Research Center of Clinical Medicine, National Cheng Kung University Hospital, College of Medicine, National Cheng Kung University, Tainan, Taiwan

**Keywords:** parechovirus A3, cytopathic effect, inflammation, neuronal diseases, infectious model

## Abstract

Human parechovirus type 3 (PeV-A3) infection has been recognized as an emerging etiologic factor causing severe nerve disease or sepsis in infants and young children. But the neuropathogenic mechanisms of PeV-A3 remain unknown. To understand the pathogenesis of PeV-A3 infection in the neuronal system, PeV-A3-mediated cytopathic effects were analyzed in human glioblastoma cells and neuroblastoma cells. PeV-A3 induced interferons and inflammatory cytokine expression in these neuronal cells. The pronounced cytopathic effects accompanied with activation of death signaling pathways of apoptosis, autophagy, and pyroptosis were detected. A new experimental disease model of parechovirus encephalitis was established. In the disease model, intracranial inoculation with PeV-A3 in C57BL/6 neonatal mice showed body weight loss, hindlimb paralysis, and approximately 20% mortality. PeV-A3 infection in the hippocampus and cortex regions of the neonatal mouse brain was revealed. Mechanistic assay supported the *in vitro* results, indicating detection of PeV-A3 replication, inflammatory cytokine expression, and death signaling transduction in mouse brain tissues. These *in vitro* and *in vivo* studies revealed that the activation of death signaling and inflammation responses is involved in PeV-A3-mediated neurological disorders. The present results might account for some of the PeV-A3-associated clinical manifestations.

## Introduction

Human parechovirus (PeV-A) is a 28-nm diameter, non-enveloped, positive-sense, signal-stranded RNA virus that belongs to the *Picornaviridae* family (1). PeV-A was first isolated from a diarrhea specimen and was originally classified as echoviruses 22 and 23 in 1956 (2). Then in view of its nucleotide sequence in replication and translation elements being different from other members of the genus *Enterovirus*, the virus was reclassified as *Parechovirus*, and echoviruses 22 and 23 were e-named PeV-A1 and PeV-A2, respectively (3–5). At present, 19 types PeV-A (PeV-A1~PeV-A19) have been reported and recognized on the basis of their viral protein 1 (VP1) sequences (6).

PeV-A is a widespread viral pathogen that affects children’s health, causing gastroenteritis, respiratory disease, or severe nerve disease in children of younger age (7). The outbreak of adult PeV-A type 3 (PeV-A3)-associated myalgia was reported in Japan, suggesting that PeV-A might be a new disease pathogen in adult (8). Owing to poor growth of PeV-A in culture as well as laborious and time-consuming technique involved, it is not routinely detected in most clinical laboratories (9, 10). Thus, the actual incidence of PeV-A infection in clinical illnesses is unknown; and more importantly, delayed diagnosis and poor management in severe conditions remain unsolved.

PeV-A3 notably results in severe diseases of the central nervous system (CNS) and neonatal sepsis (6, 11, 12), which is almost exclusively restricted to infants <3 months old (13). PeV-A3 was reported as the leading cause of CNS infection in the United States (14). Neonates have increased risk of PeV-A3 infection due probably to reduced neutralization antibody protection (15). After PeV CNS infections, some of the neonates and young children exhibited neurological sequelae and neurodevelopmental delay (16). Different from PeV-A1, PeV-A3 lacks the Arginyl-Glycyl-Aspartic acid (RGD) motif in VP1 to bind integrin receptors to the cell membrane for entering cells (17, 18). Alternatively, PeV-A3 may use an unidentified receptor for infection and change its cellular tropism, resulting in enhanced ability to spread and replicate in the CNS (19, 20).

The mechanisms causing cell death can be classified as apoptosis, necrosis, cornification, autophagy, and pyroptosis (21, 22). Toll–interleukin receptor (TIR) domain-containing adaptor-inducing interferon-β (TRIF), an adaptor for Toll-like receptor 3 (TLR3), was shown to induce apoptosis in Fas-associated *Via* Death Domain (FADD)-, caspase 3-, and caspase 8-dependent manner. Thus, TRIF links innate immunity and apoptosis pathways (23). In another cell death process, the mitochondria play an important role in controlling cell response upon virus infection (24). Autophagy features degradation of cellular components within the dying cell in autophagic vacuoles. The processes of autophagy include vacuolization, degradation of cytoplasmic contents, and slight chromatin condensation. Autophagy has been regarded as phylogenetically old process because of its involvement in the vertebrate development. Autophagy flux-involved cell death was revealed in virus-infected cells (25). Virus infection-mediated pyroptosis cell death was also reported (22). Pyroptosis is a kind of cell death accompanied with inflammation response. Several stimuli can trigger pyroptosis, such as infection, stroke, or cancer. Pyroptosis is found to be crucial for controlling microbial infections. Pathogens have developed mechanisms to inhibit pyroptosis, enhancing its persistence and ability to cause diseases. The competition between the host and the pathogen on the regulation of pyroptosis determines the life and death of the host (22).

The lack of a suitable infection model poses challenge to understanding the viral pathogenic and immunological mechanisms of PeV-A3 diseases (26). This study deciphered the mechanisms of *in vitro* and *in vivo* PeV-A3-mediated neuropathogenesis, which would be helpful for developing strategies for PeV-A3 prevention and treatment.

## Materials and Methods

### Cell Lines and Viruses

Human glioblastoma (GBM) cells (DBTRG-05MG, GBM cells) (Bioresource Collection and Research Center, BCRC-60380, Hsinchu, Taiwan) were maintained in RPMI-1640 medium (Gibco, #31800022, Waltham, MA, USA). Vero cells (American Type Culture Collection (ATCC), CCL-81, Manassas, VA, USA) and human neuroblastoma cells (IMR-32 cells, ATCC, CCL-127, Manassas, VA, USA) were cultured in MEM/EBSS medium (HyClone, #SH30008.02, Logan, UT, USA). Cell culture mediums were supplemented with 10% fetal bovine serum (FBS) (Gibco, A4766801, Waltham, MA, USA) and penicillin-streptomycin (P/S) (Gibco, #15140122, Waltham, MA, USA). PeV-A3 was from a virology group, Department of Microbiology, Kaohsiung Veterans General Hospital (27). PeV-A3 was propagated in Vero cells. The full genome sequence of PeV-A3 (strain VGHKKS-2007 accession #KM986843) has been characterized (28).

### Immunofluorescence Assay

For cell staining, cells were plated at 1 × 10^4^ cells/well in 96-well plates and incubated overnight. The cells were then infected with PeV-A3 at multiplicity of infection (MOI) = 5. After 8- and 24-h infection, the cells were fixed with 1:1 Methanol/Ethanol solution (Sigma-Aldrich, St. Louis, MO, USA) for 15 min. Cells were incubated with blocking buffer [10% skim milk in phosphate-buffered saline (PBS)] and incubated with anti-PeV VP0 polyclonal antibody at 4°C overnight (LTK BioLaboratories, Taoyuan, Taiwan) (10, 29). Then, the IRDye^®^ 800CW goat anti-rabbit IgG secondary antibody was added into the cells (Li-COR, #926-32211, Lincoln, NE, USA), followed by 2-h incubation. Images of immunofluorescence assay were captured, and the fluorescence intensity of PeV-A3-infected cells were quantified using Odyssey image system (Li-COR, Lincoln, NE, USA).

### RNA Extraction, Reverse Transcriptase PCR, and Quantitative Real-Time PCR

Total RNA in cells or tissues were extracted first with 1 ml of TRIzol™ reagent (Invitrogen, #15596018, Waltham, MA, USA) and then with 1-bromo-3-chloropropane (BCP) (Sigma-Aldrich, B9673, St. Louis, MO, USA). The emulsion was centrifuged, and the aqueous phase was transferred to a fresh tube. The RNA was precipitated with 500 µl of isopropanol (Sigma-Aldrich, #278475, St. Louis, MO, USA) and pelleted by centrifugation at 12,000 × rpm at 4°C for 15 min. RNA pellet was washed with 1 ml of 75% ethanol and then centrifuged at 12,000 × rpm at 4°C for 10 min. After removal of supernatant, RNA pellet was air-dried for 10 min in a laminar flow hood and resuspended in nuclease-free water.

The RNA concentration was measured using a spectrophotometer (Eppendorf, Hamburg, Germany). For reverse transcriptase, cDNA was synthesized using 50 mM of random primer with 2 μg of total RNA in a total reaction volume of 20 μl using Superscript III reverse transcriptase method (Invitrogen, 18080085, Waltham, MA, USA). qPCR amplification involved 4 ng of cDNA in Fast SYBR™ Green Master Mix (Thermo Fisher, #4385612, Waltham, MA, USA) with 3 μM of primers (Genomics, New Taipei City, Taiwan) (**Table S1**). qPCR was performed at 95°C for 3 min for 1 cycle and then 95°C for 20 s and 60°C for 30 s for 40 cycles. A final melting curve stage was performed from 60°C to 95°C using ABI StepOnePlus Real-Time PCR System (Life Technologies, Waltham, MA, USA).

### RT^2^ Profiler PCR Array

The cDNA was synthesized from 2 μg of total RNA with 50 mM of random primer in a total reaction volume of 14 μl using RT^2^ First Strand Kit (Qiagen, #330401, Hilden, Germany). The cDNA quality control of genomic DNA contamination (GDC), first-strand synthesis (RTC), and real-time PCR efficiency (PPC) were confirmed according to the manufacturer’s instruction. Finally, 25 μl of PCR component mixture in each well was used for the analysis of human cell death pathway using RT^2^ Profiler PCR plate array (Qiagen, #330231, Hilden, Germany) in ABI StepOnePlus Real-Time PCR System.

### Cell Viability Assay

GBM cells (1 × 10^4^ cells/well) were grown in 96-well plates overnight and then infected with PeV-A3 at MOI = 0.1, 1, and 10. After infection for 6 and 24 h, cells were incubated with 10 μl of WST-1 reagent (Roche-Sigma-Aldrich, #116448807001, St. Louis, MO, USA) for 2 h. The absorbance at 450 nm was monitored, and the reference wavelength was set at 620 nm using a microplate spectrophotometer (Epoch, BioTek, Winooski, VT, USA).

### JC-10 Mitochondrial Membrane Potential Assay

GBM and IMR-32 cells (5 × 10^5^ cells/well) were grown in six-well plates overnight and then infected with PeV-A3 at MOI = 1 for 24 h. After infection, cells were harvested for JC-10 analysis (Abcam, #ab112133, Cambridge, England). Cells were suspended in 500 µl of JC-10 solution and incubated at 37°C for 45 min, protected from the light. Then, cells with fluorescence were analyzed using flow cytometry (BD FACSCalibur Franklin Lakes, NJ, USA). Fluorescence-activated cell sorting (FACS) analysis displayed cell subsets, which were estimated with respect to populations selected on the basis of membrane potential change. Apoptotic cells lost the mitochondria inner membrane potential, and the monomeric form of JC-10 was released into the cytoplasm. Cells displayed red fluorescence loss and turned green. The FL1 channel of FACS was used for detecting green fluorescent monomeric signal in apoptotic cells, while the FL2 channel was used for the detecting orange fluorescent aggregated signal in healthy cells.

### Western Blotting Analysis

Cells and mouse brain tissue lysates were lysed in protein lysis buffer (2% sodium dodecyl sulfate (SDS) and 50 mM of Tris-HCl, pH = 7.5) containing protease and phosphatase inhibitors (Thermo Fisher, #78442, Waltham, MA, USA). The lysates were homogenously sonicated, separated in 10% or 12% SDS–polyacrylamide gel electrophoresis (PAGE), and finally transferred to polyvinylidene difluoride (PVDF) membranes (Millipore, IPVH00010, Burlington, MA, USA). PVDF membranes were treated with 10% milk blocking buffer and incubated with primary antibody in 1:2,000 dilution in 5% milk/TBST at 4°C overnight, Antibodies used included anti-PeV VP0 (10), anti-caspase 3 (Cell Signaling, #9662, Danvers, MA, USA), anti-caspase 7 (GeneTex, GTX123679, Hsinchu City, Taiwan), anti-PARP (Cell Signaling, #9532, Danvers, MA, USA), anti-caspase 8 (Cell Signaling, #9746, Danvers, MA, USA), anti-caspase 9 (Cell Signaling, #9508, Danvers, MA, USA), anti-FASLG (GeneTex, GTX31191, Irvine, CA, USA), anti-CD40 (GeneTex, GTX101447, Irvine, CA, USA), anti-LC3 A/B (Cell Signaling, #4108, Danvers, MA, USA), anti-p62 (Cell Signaling, #16177, Danvers, MA, USA), anti-beclin 1 (Cell Signaling, #3495, Danvers, MA, USA), anti-phospho-NF-κB p65 (Ser536) (Cell Signaling, #3033, Danvers, MA, USA), anti-NLRP3 (Cell Signaling, #13158, Danvers, MA, USA), anti-IL1β (Cell Signaling, #12242, Danvers, MA, USA), anti-RIPK1 (R&D, MAB3585, Minneapolis, MN, USA), anti-RIPK3 (R&D, MAB7604, Minneapolis, MN, USA), anti-GSDMD (GeneTex, GTX135366, Hsinchu City, Taiwan), anti-β-actin (ProteinTech, #66009-1-Ig, Rosemont, IL, USA), and anti-GAPDH (ProteinTech, #60004-1-Ig, Rosemont, IL, USA). Secondary antibodies conjugated with horseradish peroxidase (HRP)-anti mouse (LEADGENE, 20112, Tainan, Taiwan) and HRP-anti rabbit (LEADGENE, #20202, Tainan, Taiwan) were incubated at room temperature 1 h. The images were acquired from biospectrum imaging system (UVP Analytik Jena, Jena, Germany) by using WesternBright ECL kit (Advansta, K-12045-D20, Menlo Park, CA, USA).

### Virus Infection and Disease Symptoms Observed in Neonatal Mice

C57BL/6 mice were purchased from the National Laboratory Animal Center (NARLabs, Taipei, Taiwan). This study was approved by the Institutional Animal Care and Use Committee or Panel (IACUC) in Kaohsiung Veterans General Hospital, Taiwan (IACUC Approval No. VGHKS-2018-2021-A016). According to the reported method of intracranial injection of virus (30), 3-day-old C57BL/6 neonatal mice were intracranially inoculated with PeV-A3 (5 × 10^5^ pfu/mouse) into the cerebral lateral ventricles using Hamilton 1700 Series Syringes (Hamilton, 80000, Reno, NV, USA). PBS inoculation on mice was the control. Survival rate, clinical scores, and body weight were then monitored daily after inoculation. Clinical scores 0–5 indicated the severity level of disease symptoms: 0, healthy; 1, reduced mobility; 2, reduced body weight; 3, forelimb or hindlimb weakness; 4, forelimb or hindlimb paralysis; and 5, death.

### Immunohistochemistry

Paraffin-embedded brain tissues from PeV-A3 mice and PBS mice were cut and mounted on coated slides. The sections were de-paraffinized in xylene and rehydrated through graded alcohol solutions, and antigen retrieval in sodium citrate buffer at 100°C for 20 min was performed. The immunohistochemistry (IHC) procedures of Novolink Polymer Detection Systems (Leia #RE7150-K, Wetzlar, Germany) were modified. Brain sections were incubated with Protein Block buffer for blocking 15 min and then incubated overnight at 4°C with rabbit polyclonal antibody anti-PeV-VP0 and rabbit IgG isotype control (Thermo Fisher, #02-6102, Waltham, MA, USA). After being rinsed with PBS (Biological Industries #11-223-1M Cromwell, CT, USA), the slides were incubated with secondary antibody goat anti-rabbit IgG Alexa Flour 488 (1:500 dilution, Thermo Fisher, #A32731, Waltham, MA, USA) for 1 h at room temperature. The slides were mounted after DAPI staining (1:5,000 dilution, Thermo Fisher #62248, Waltham, MA, USA) for 30 min. The hippocampus and cortex regions were observed under an inverted fluorescence microscope (Olympus, Tokyo, Japan).

### Statistical Analysis

Statistical analysis was performed using Prism 9 (GraphPad, San Diego, CA, USA). Student’s t-test or Mann–Whitney U test were used for quantitative parameters. Survival rates were analyzed using Kaplan–Meier curve. Results were expressed as mean ± SD. *p*-Value of <0.05 was considered to be significant.

## Results

### Parechovirus Type 3 Replication in Human Glioblastoma and Neuroblastoma Cells

**Figure 1:**
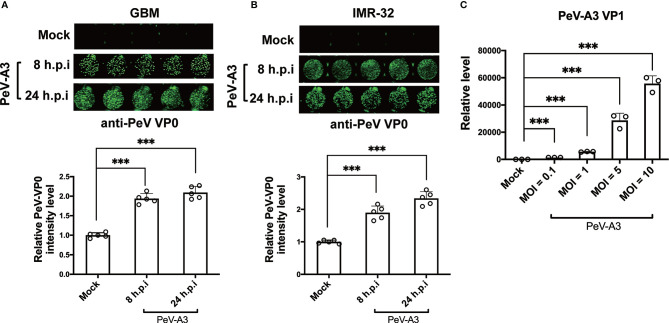
Replication of PeV-A3 infection in GBM and IMR-32 cells. **(A)** GBM and **(B)** IMR-32 cells were infected with PeV-A3 at MOI = 5 for 8 and 24 h. Cells were then stained with anti-PeV AP0 antibody followed by IRDye^®^ 800CW goat anti-rabbit IgG secondary antibody. The cell images of immunofluorescent assay were captured (top panels) using Odyssey image system. Fluorescence intensities of PeV-A3-infected cells were quantified (bottom panels). Data are mean ± SD of five independent experiments. Student’s t-test, ***p < 0.001. **(C)** RT-qPCR of PeV-A3 VP1 expression in GBM cells infected with PeV-A3 at MOI = 0.1, 1, 5, and 10 for 24 h. Data are mean ± SD. Student’s t-test, ***p < 0.001 compared with mock controls. PeV-A3, parechovirus type 3; GBM, glioblastoma; MOI, multiplicity of infection; VP1, viral protein 1.

GBM cells and neuroblastoma cells were used in research on neurotropic virus infections such as ZIKA virus and enterovirus 71 (31–34). Therefore, this study used human GBM cells (DBTRG-05MG) and human neuroblastoma cells (IMR-32) to decipher the neuropathic effects of PeV-A3. These two cell lines were infected with PeV-A3 at MOI = 5 for 8 and 24 h, followed by immunofluorescence analysis conducted with anti-PeV VP0 antibody. Green fluorescence indicated VP0 expression in PeV-A3-infected cells (**Figures 1A, B**, top panels). The fluorescence level was further quantified. Comparison with mock controls and results at 8 h post infection (h.p.i) revealed an increased VP0 expression level detected at 24 h.p.i. (**Figures 1A, B**, bottom panels). The viral genome in GBM cells infected with PeV-A3 at MOI = 0.1, 1, 5, and 10 was measured. A gradual viral load-dependent increase in PeV-A3 VP1 mRNA level was observed (**Figure 1C**). Taken together, these results showed susceptibility of GBM and neuroblastoma cells to PeV-A3 infection.

### Induction of Type I and Type III IFN by Parechovirus Type 3 and Inhibition of Parechovirus Type 3 Replication by IFN Treatment

**Figure 2:**
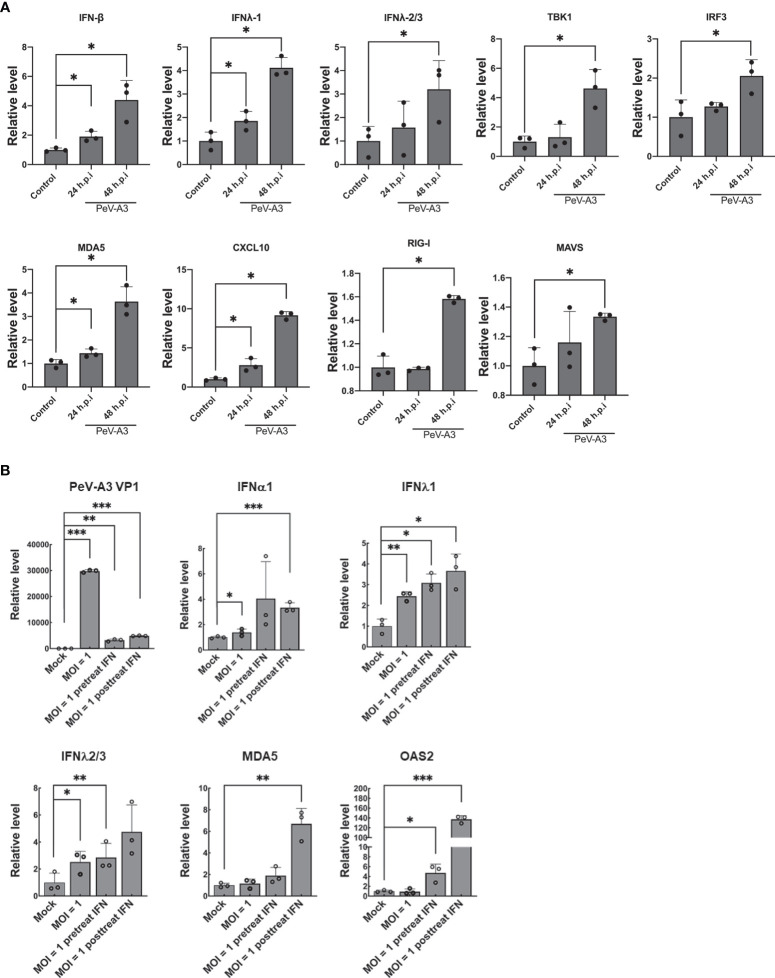
PeV-A3 infection activated cellular antiviral innate immunity. **(A)** GBM cells were infected with PeV-A3 at MOI = 1 for 24 and 48 h. mRNA levels of antiviral genes IFNβ, IFN λ1, IFNλ2/3, TBK1, IRF3, MDA5, CXCL10, RIG-I, and MAVS were confirmed using RT-qPCR. The level of transcripts was normalized by GAPDH. Data are mean ± SD of three independent experimental samples. Student’s t-test, *p < 0.05 compared with mock controls. **(B)** Efficacy of type I IFN against PeV-A3. IFN-α2 measuring 100 IU/ml was pretreated or posttreated in GBM cells for 24 h. GBM cells were infected with PeV-A3 at MOI = 1 for 24 h. Expression of mRNA of IFNα1, IFN λ1, IFNλ2/3, MDA5, and OAS2 was measured using RT-qPCR and normalized to GAPDH. Data are mean ± SD of three independent samples. Student’s t-test, *p < 0.05; **p < 0.01, ***p < 0.001 compared with mock controls. PeV-A3, parechovirus type 3; GBM, glioblastoma; MOI, multiplicity of infection.

During viral infection, cellular type I and type III IFN antiviral responses are evoked to restrict virus replication (35). In the CNS, type I IFN protects neurons from prion infection (36). To clarify the PeV-A3-induced antiviral response in GBM, we measured the IFN and associated antiviral protein expression after infection for 24 and 48 h. RT-qPCR results showed significant induction of the determined genes, which were IFN-β, type III IFN (IFNλ-1, IFNλ-2/3), TANK-binding kinase 1 (TBK1), interferon regulatory factor 3 (IRF3), melanoma differentiation-associated protein 5 (MDA5), C-X-C motif chemokine ligand 10 (CXCL10), retinoic acid-inducible gene I (RIG-I), and mitochondrial antiviral-signaling protein (MAVS) transcripts (**Figure 2A**). These findings suggested that PeV-A3 activated cellular antiviral responses in GBM cells.

We further investigated type I IFN activity against PeV-A3 in GBM. Cells were infected with PeV-A3 at MOI = 1 for 48 h after being pretreated or posttreated with type I IFN 100 IU/ml for 24 h. RT-qPCR results showed reduction of viral replication along with induction of type I and type III IFN and ISGs (MDA-5 and 2′-5′-Oligoadenylate Synthetase 2, OAS2) (**Figure 2B**). PeV-A3-activated antiviral responses and exogenous type I IFN treatment-suppressed PeV-A3 suggest a typical virus–host interaction in GBM cells.

### Parechovirus Type 3 Caused Cytopathic Effect in Neuronal Cells

**Figure 3:**
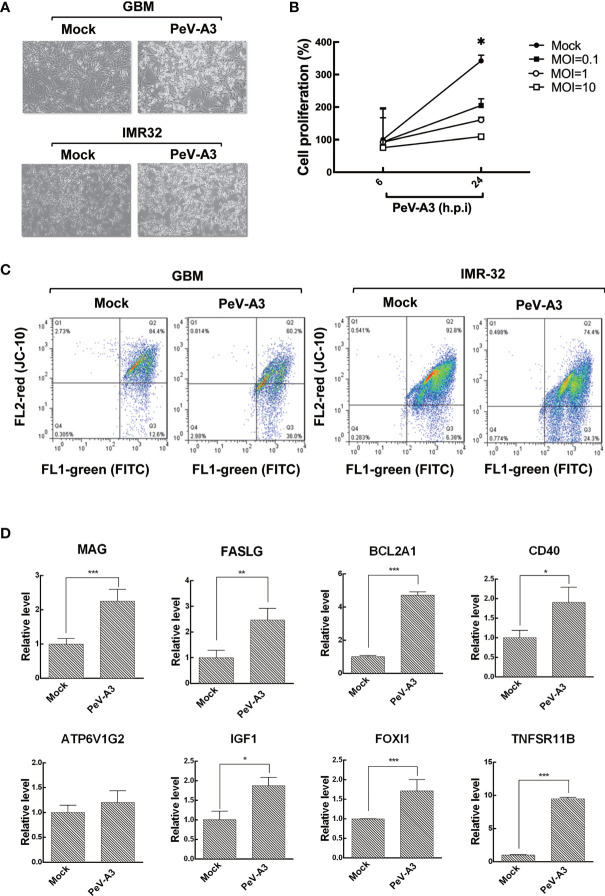
PeV-A3 induced cell death. **(A)** CPE observation of GBM and IMR-32 cells infected with PeV-A3 at MOI = 1 for 24 h. **(B)** WST-1 assay was conducted in GBM cells infected with PeV-A3 at MOI = 0.1, 1, and 10 for 6 and 24 h. Cell viability was detected in a microplate ELISA reader at absorbance OD 450 nm and reference wavelength OD 620 nm. **(C)** Flow cytometry analysis of JC-10 mitochondrial membrane potential detection in PeV-A3-infected GBM and IMR-32 cells. Cells located in Q2 area are healthy. Cells located in Q3 are damaged, showing loss of mitochondrial inner membrane potential. **(D)** RT-qPCR analysis of genes identified using RT^2^ array. RT-qPCR was conducted in PeV-A3-infected GBM cells and mock controls. Data are mean ± SD of three independent experiments. Student’s t-test, *p < 0.05; **p < 0.01; ***p < 0.005 compared with mock controls. PeV-A3, parechovirus type 3; CPE, cytopathic effect; GBM, glioblastoma; MOI, multiplicity of infection; OD, optical density.

Although type I IFN response was elicited by PeV-A3 in GBM cells, the host antiviral activity failed to efficiently restrict this virus, with both GBM and IMR-32 cells showing cytopathic effect (CPE) morphology upon PeV-A3 infection at MOI = 1 for 24 h (**Figure 3A**). The WST-1 assay of GBM cells proliferation was impaired by PeV-A3, revealing a decreased cell growth in a viral load-dependent manner (**Figure 3B**). This study further investigated whether PeV-A3 infection caused mitochondrial membrane potential change using cationic, lipophilic JC-10 dye staining in GBM and IMR-32 cells. Compared with 12.6% and 6.38% of mock-infected GBM and IMR-32 cells, respectively showing JC-10 background signaling, 36% and 24.3% of PeV-A3-infected GBM and IMR-32 cells respectively exhibited mitochondrial membrane potential loss, becoming apoptotic cells (**Figure 3C**). Collectively, these findings revealed a pathogenic effect of PeV-A3 in neuronal cell types.

To understand the expression pattern of cell death-related genes in PeV-A3-infected GBM cells, the human RT^2^ profiler PCR array was conducted (**Table S2**). Results showed that PeV-A3-infected GBM cells expressed death protein genes at least twofold upregulated over mock controls including myelin-associated glycoprotein (MAG), Fas ligand (FASLG), BCL2-related protein A1 (BCL2A1), ATPase H+ Transporting V1 Subunit G2 (ATP6V1G2), insulin-like growth factor 1 (IGF1), Forkhead box I1 (FOXl1), and tumor necrosis factor receptor superfamily, member 11b (TNFSR11B) (**Figures S1A, B**). The expression level of these genes was further confirmed using RT-qPCR analysis, which showed a significant induction, compared with mock controls (**Figure 3D** and **Table S3**). Hence, results obtained evidenced that PeV-A3 infection triggered cell death-associated gene transcript in GBM cells.

### Parechovirus Type 3 Activated Cell Death Signaling Pathways in Glioblastoma Cells

**Figure 4:**
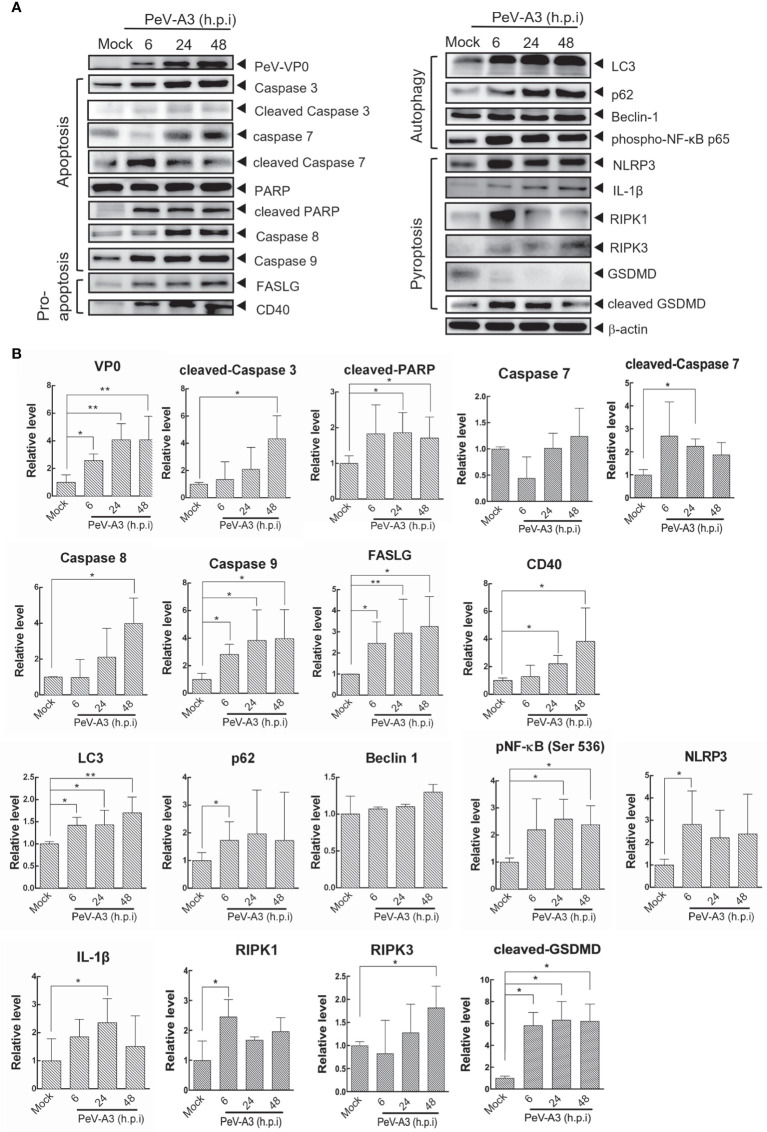
Cell death signaling analysis in PeV-A3-infected GBM cells. **(A)** GBM cells were infected with PeV-A3 at MOI = 1 for 6, 24, and 48 h. Cell extracts were employed to analyze cell death signaling pathways by Western blotting using anti-PeV VP0, anti-caspase 3, anti-PARP, anti-caspase 8, anti-caspase 9, anti-FASLG, anti-CD40, anti-LC3, anti-p62, anti-beclin 1, anti-phospho-NF-κB p65 (Ser536), anti-NLRP3, anti-IL1β, anti-RIPK1, anti-RIPK3, anti-GSDMD, and anti-β-actin. **(B)** Quantified expression of different proteins was normalized to β-actin. Data are mean ± SD of three independent experiments. Student’s t-test, *p < 0.05; **p < 0.01 compared with mock controls. PeV-A3, parechovirus type 3; GBM, glioblastoma; MOI, multiplicity of infection.

Programmed cell death is an important machinery in host’s defense to limit the replication of invading intracellular pathogens. Types of viral-mediated cell death included apoptosis, programmed necrosis, autophagy, and pyroptosis; the events promote the formation of effective long-term host immune inflammation and innate responses (23, 37). After detection of PeV-A3-mediated cell death, death signaling was analyzed to understand the cellular activity in response to PeV-A3 infection. Immunoblotting of signaling proteins was conducted in GBM cells infected with PeV-A3 for 6, 24, and 48 h. The protein level was also quantified and statistically analyzed. The time dependently increased PeV-A3 VP0 protein level revealed the spread of viral infection status in GBM cells. We detected significant increase in signaling protein level underlying apoptosis and pro-apoptosis pathways, such as activated caspase-3, caspase-7, PARP, caspases 8, caspase 9, FASLG, and CD40, after PeV-A3 infection in a time-dependent manner (**Figure 4A** left panel and **Figure 4B**).

Autophagy is a process that consigns cytoplasmic components to lysosomes for degradation. Autophagy is usually associated with cell death. Autophagy-associated cell death depends largely on the physical conditions (37, 38). Autophagy-induced NFκB activity was also reported, which could be targeted by viral infection (39, 40). This study found significant accumulation of autophagic factors LC3 and p62 and a slight increase in the beclin-1 level of GBM cells during PeV-A3 infection periods. The induced phosphorylated NF-κB p65 Ser536 activity was also detected. The above results suggested that PeV-A3 infection-associated autophagy influx is possibly involved in PeV-A3-triggered cell death (**Figure 4A**, right panel, and **Figure 4B**). Since PeV-A3-caused neuronal cell death was revealed (**Figure 3C**), we also analyzed the apoptosis and pro-apoptosis and autophagy signaling responses in IMR-32 cells with PeV-A3 infection. Compared with that of mock controls, the increased protein level of activated caspase 3, CD40, FASLG, LC3, and p62 was detected (**Figure S2**).

Pyroptosis is a type of cell death accompanied with inflammation and is essential for limiting pathogen infections. Infections stimulate pyroptosis activation and formation of NLR family pyrin domain containing 3 (NLRP-3) inflammasome, resulting in processing and activation of caspase 1 for expression of inflammatory cytokines interleukin 1β (IL-1β) and IL-18 (41, 42). In the signal pathways of NLRP3-mediated pyroptosis, IL-1β and receptor-interacting protein (RIP) family of serine-threonine kinases, RIPK1 and RIPK3, Gasdermin-D (GSDMD) were detected in PeV-A3-infected GBM cells (**Figure 4A**, right panel, and **Figure 4B**). This study detected activation of death signaling of apoptosis, autophagy, and pyroptosis as early as 6 h.p.i. in GBM cells, suggesting a fast pathogenic response promoted upon PeV-A3 replication. Together, these results suggested that PeV-A3 induced multiple cell death signaling pathways.

### Parechovirus Type 3 Induced Inflammatory Cytokines

**Figure 5:**
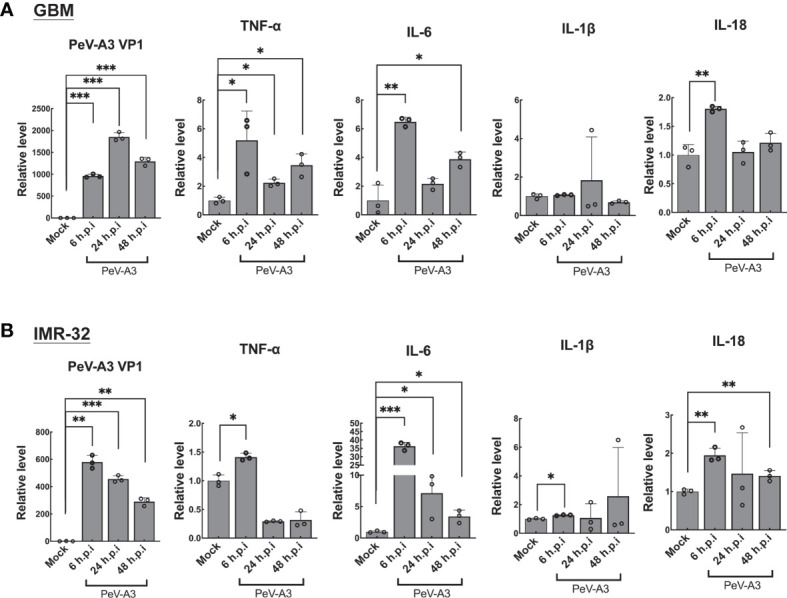
PeV-A3 induced expression of pyroptosis-associated inflammatory cytokines. **(A)** GBM cells and **(B)** IMR-32 cells were infected with PeV-A3 at MOI = 1 for 6, 24, and 48 h. TNF-α, IL-6, IL-1β, and IL-18 were analyzed using RT-qPCR. Data are mean ± SD of three independent experiments. Student’s t-test *p < 0.05; **p < 0.01; ***p < 0.001 compared with mock controls. PeV-A3, parechovirus type 3; GBM, glioblastoma; MOI, multiplicity of infection.

**Figure 6:**
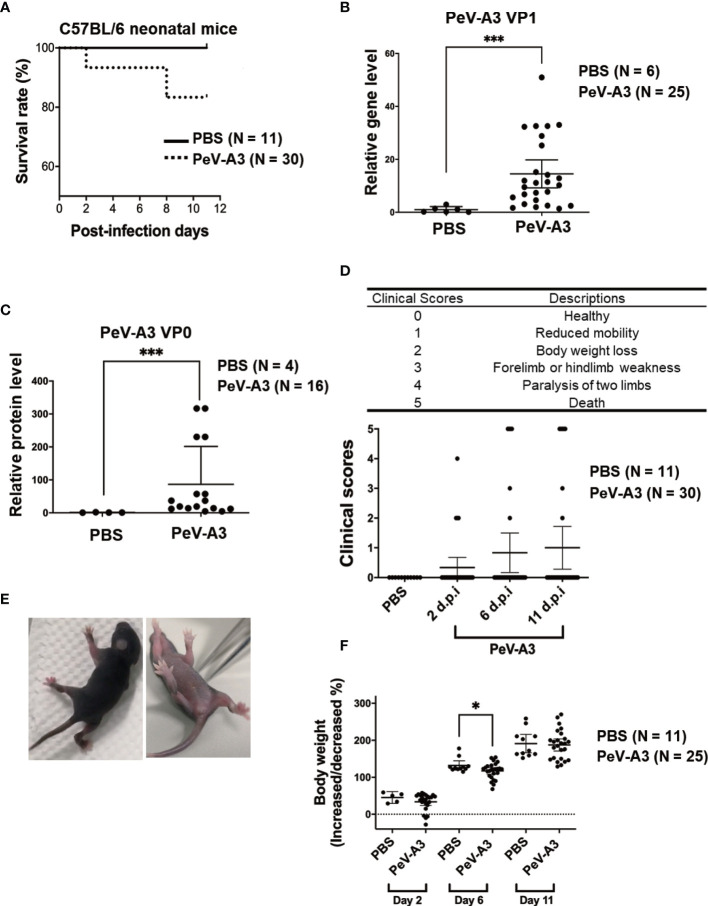
PeV-A3 caused neuronopathic effects in C57BL/6 neonatal mice. **(A)** Three-day-old C57BL/6 mice were intracranially inoculated with PeV-A3 (5 × 10^5^ pfu/mouse) or PBS as mock controls. The survival rate was monitored from days 0 to 11 after inoculation (PBS N = 11, PeV-A3 N = 30). Mann–Whitney U test, ***p < 0.001 compared with PBS controls. **(B)** Eleven days after inoculation, the mice were sacrificed, and total RNA was isolated from brain tissues for PeV-A3 VP1 mRNA detection. Data are mean ± SD, PBS group, N = 6; PeV-A3 group, N = 25. Independent two-sample t-test, *p* < 0.001 compared with PBS controls. **(C)** Mouse brain tissue protein lysates were subjected to immunoblotting with anti-PeV-VP0 and anti-GAPDH; the relative expression of PeV-A3 VP0 protein was normalized using GAPDH. PBS controls, N = 4, PeV-A3 N = 16. Independent two-sample t-test, ***p < 0.001 compared with PBS mock control. **(D)** Description of clinical symptoms corresponded with clinical scores: 0, healthy; 1, reduced mobility; 2, reduced body weight; 3, forelimb or hindlimb weakness; 4, forelimb or hindlimb paralysis; and 5, death. Clinical scores were monitored daily. Clinical score in mice after inoculation at days 2, 6, and 11 were shown. Data are mean ± SD. PBS group, N = 11, PeV-A3 group, N = 30. Independent two-sample t-test. **(E)** Photo images showed PeV-A3 infection caused paralysis in two limbs at day 2 after inoculation in C57BL/6 mice. **(F)** Changes in body weight were measured daily. Changes in mice weight after inoculation at days 2, 6, and 11 are shown. Data are mean ± SD, PBS control group, N = 11; PeV-A3, N = 25. Independent two-sample t-test, *p < 0.05 compared with PBS controls. PeV-A3, parechovirus type 3; PBS, phosphate-buffered saline; VP1, viral protein 1.

In view of PeV-A3 infection-triggered signaling activation of autophagy and pyroptosis, this study then accessed autophagy- and pyroptosis-mediated inflammatory cytokine expression responses in GBM and IMR-32 cells. These cells were infected with PeV-A3 for 6, 24, and 48 h, followed by the induction of TNF-α, IL-6, IL-1β, and IL-18 measured using RT-qPCR. Results showed similar transcript patterns in GBM (**Figure 5A**) and IMR-32 cells (**Figure 5B**), where inflammatory cytokines, TNF-α, IL-6, IL-1β, and IL-18 transcripts, were significantly induced at 6 h.p.i. and then declined at 24 and 48 h.p.i. These data suggested that, consistent with early activation of death signaling by PeV-A3 shown in **Figure 6**, an early response of inflammatory transcript in GBM cells was also detected. The reduction of these inflammatory genes might be due to impaired cells’ viability and low activity of host gene transcription. Collectively, the above findings revealed the neuropathogenic mechanism of PeV-A3 *in vitro*.

### Parechovirus Type 3 Caused Neuropathic Symptoms in Neonatal Mice

The above findings evidenced that PeV-A3 infection inhibited cell growth and activated cell death pathways including apoptosis, autophagy, and pyroptosis *in vitro*. To investigate the PeV-A3-induced neuropathic disease *in vivo*, we established a PeV-A3 infection animal model by using 3-day-old C57BL/6 mice. Neonate mice were intracranially inoculated with PeV-A3 at 5 × 10^5^ pfu/mouse, while littermates that received PBS injection served as mock controls. After inoculation, the survival rate was monitored from day 0 to day 11. Results showed approximately 20% mortality in PeV-A3-infected mice (**Figure 6A**).

After infection for 11 days, the mice were sacrificed, and total RNA was extracted from the brain tissues to analyze the PeV-A3 VP1 mRNA expression. RT-qPCR results showed significant expression of PeV-A3 RNA in mouse brain as compared with PBS control (**Figure 6B**). The PeV-VP0 protein expression in each mouse brain was also detected by immunoblotting, and the protein level was quantified (**Figure 6C**). The PeV-A3 viral RNA and protein expression indicated replication of PeV-A3 in the neonatal mouse brain.

According to the severity of physical condition of the mice (health, reduced mobility, body weight loss, limb weakness, paralysis, and death; **Figure 6D**, top panel), clinical symptoms observed from days 0 to 11 post injection were scored. Days 2, 6, and 11 showed progression of disease with increasing clinical scores in PeV-A3-infected mice (**Figure 6D**, bottom panel). The images showed mice with limb paralysis or moribund (**Figure 6E**). These results suggested that PeV-A3 induced strong neurovirulence in C57BL/6 mice. Changes in daily body weight in surviving PeV-A3-infected mice were measured. The findings showed lower average body weight in PeV-A3-infected C57BL/6 neonatal mice at days 2, 6, and 11 after inoculation as compared with PBS control mice (**Figure 6F**). This infection animal model demonstrated susceptibility of neonatal C57BL/6 mice to PeV-A3 infection, which caused neuropathic symptoms and decreased survival rate.

### Parechovirus Type 3 Infection in the Hippocampus and Cortex of Brain

**Figure 7:**
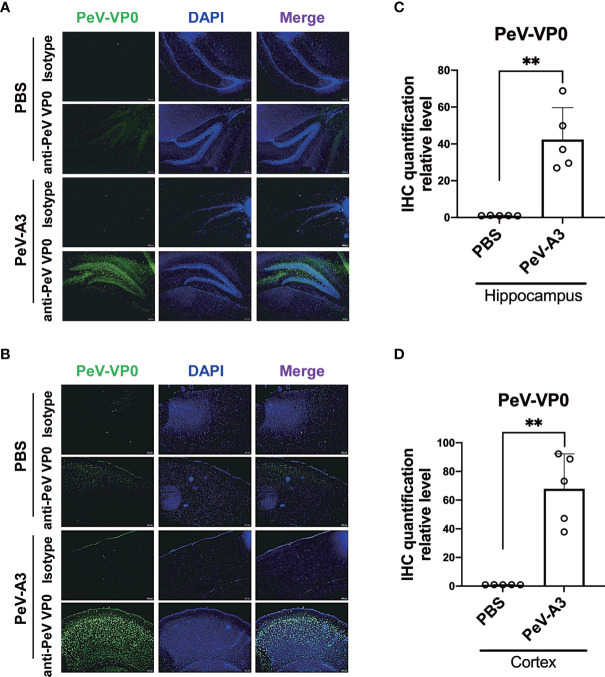
Fluorescence immunohistochemistry assay (IHC) of PeV-A3 infection in neonatal mouse brain. **(A, B)** Immunohistochemically staining for PeV-VP0 in the hippocampus and cortex section (PBS control, N = 5; PeV-A3, N = 5). Scale bar = 100 µm. **(C, D)** Quantified PeV VP0 protein level of the IHC assay of each mouse brain tissue are shown as scattered plots. Data are mean ± SD. Student’s t-test, **p < 0.01 compared with PBS. PeV-A3, parechovirus type 3; PBS, phosphate-buffered saline.

PeV-A3 RNA and protein were detected in the brain extracts of infected mice. We further conducted IHC analysis to understand whether the hippocampus and cortex of brain regions were infected by PeV-A3. The result showed that the PeV-VP0 expression was detected in the hippocampus and cortex in the PeV-A3-infected mice, but not in the PBS control mice (**Figures 7A, B**). Quantification data showed a significant high level of PeV-VP0 expression in the hippocampus and cortex; compared with PBS control, nearly 40-fold and 70-fold protein level increases were detected in the hippocampus and cortex sections, respectively (**Figures 7C, D**). Taken together, these results suggest that PeV-A3 directly infected hippocampus and cortex regions of the neonatal mouse brain.

### Parechovirus Type 3 Induced Inflammatory Cytokines and Death Signaling in Mouse Brain Tissues

**Figure 8:**
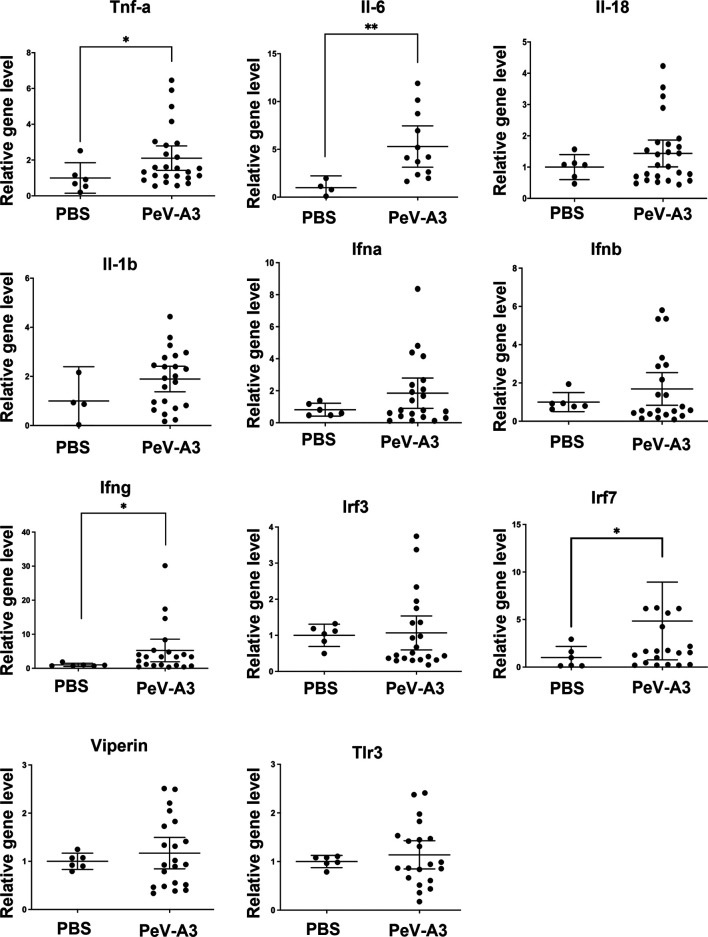
PeV-A3 induced inflammatory cytokine expression and signal transduction in mouse brain tissues. mRNA expression of inflammatory cytokines (Tnfa, Il-6, Il-8, and Il-1b) and interferon signature genes (Ifna, Ifnb Ifng Irf3, Irf7, Viprin, and Tlr3) in PeV-A3-infected C57BL/6 mouse brain tissues were measured and normalized using HPRT. Data are mean ± SD, PBS group, N = 4-6, PeV-A3 group, N = 12–25. Independent two-sample t-test, *p < 0.05; **p < 0.01 compared with PBS. PeV-A3, parechovirus type 3; PBS, phosphate-buffered saline.

**Figure 9:**
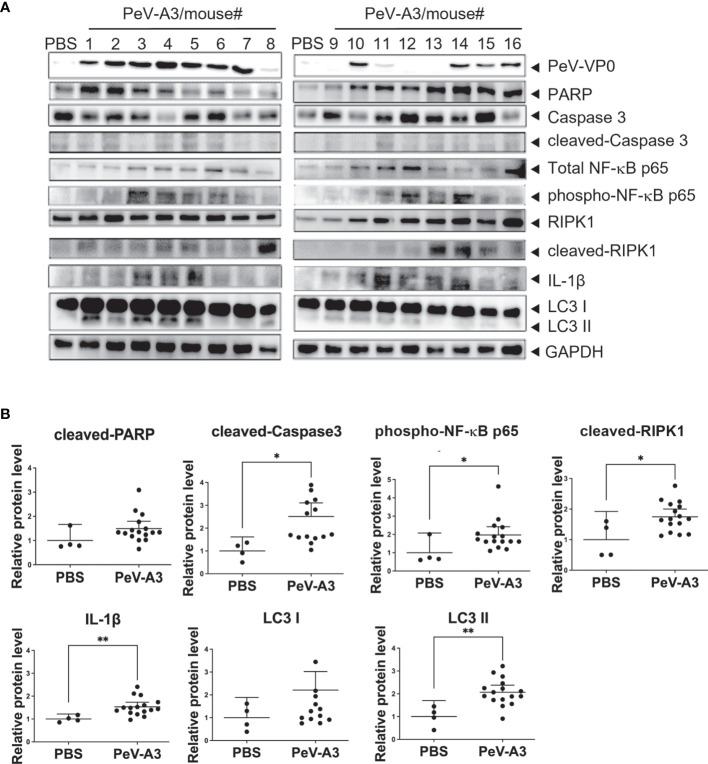
PeV-A3 induced signal transduction proteins in mouse brain tissues. **(A)** Immunoblotting of PARP, caspase 3, LC3, phospho-NF-κB p65 (Ser536), IL-1β, and RIPK1 in two PBS control and 16 PeV-A3 mouse brain tissues (#1-#16). **(B)** Quantification of protein expression level of PeV-A3-infected mice and PBS mock controls. Data are mean ± SD, PBS group N = 4, PeV-A3 group N = 16. Independent two-sample t-test, *p < 0.05 **p < 0.01 compared with PBS mock controls. PeV-A3, parechovirus type 3; PBS, phosphate-buffered saline.

PeV-A3 caused neurological symptoms in neonatal mice. Mouse brain tissues were examined to understand whether the present *in vitro* findings of PeV-A3-triggered inflammation and whether cell death signaling activation also occurred in the infected mice. RT-qPCR results showed a higher transcript level of inflammatory cytokines (Tnfa, Il-6, Il-8, and Il-1b) and interferon signature [Ifna, Ifnb, Ifng, interferon regulatory factor 3 (Irf3), Irf7, Viprin, and Toll-like receptor 3 (Tlr3)] in PeV-A3-infected brain tissues compared with PBS controls (**Figure 8**). Furthermore, the protein extract from each mouse brain tissue was employed to analyze death signaling; the quantified immunoblots demonstrated that brain tissues of PeV-A3-infected mice expressed a higher protein level of activated cleaved-caspase 3, cleaved-PARP, LC3, phospho-NF-κB p65, IL-1β cytokine, and RIPK1 than did PBS controls (**Figures 9A, B**). These results suggested that PeV-A3 infection induced inflammatory cytokine expression and activated apoptosis, autophagy, and pyroptosis signaling pathways in neonatal mice, which might be associated with the neurovirulence of PeV-A3.

## Discussion

Viral meningoencephalitis is a life-threatening illness in humans that can be caused by different viruses. PeV-A3 has been known as a severe viral infection in neonates and young infants, causing encephalitis- or sepsis-like diseases (6, 43–45). Despite accumulated clinical evidences of the pathovirulence of PeV-A3, the pathogenic mechanisms of PeV-A3 infection remain unclear. Thus, to understand the infection of PeV-A3 in the CNS, this study used a clinically isolated PeV-A3 strain to establish infection models *in vitro* and *in vivo*, with human neuronal cells (GBM and IMR-32) and C57BL/6 neonatal mice, respectively. Results showed that PeV-A3 induced cell damages and triggered expression of inflammatory cytokines along with activation of death signaling pathways of apoptosis, autophagy, and pyroptosis. In the *in vivo* neonatal mouse model, the PeV-A3-infected mice showed body weight loss and CNS disease symptoms, such as limb weakness or paralysis, with approximately 20% mortality. PeV-A3 infection in the hippocampus and cortex regions was detected. PeV-A3 replication, inflammatory cytokine expression, and cell death signal activation were demonstrated in the mouse brain tissue. These results obtained in neonatal mice might partly interpret the pathogenesis of PeV-A3 (46).

Type I and type III IFN responses play a pivotal role in host antiviral machinery; they elicit expression of cellular antiviral proteins to restrict virus replication (35). The present *in vitro* infection models revealed that PeV-A3 infection evoked a cellular type I and type III IFN antiviral activity and inflammatory response in GBM and IMR-32 cells. These findings echoed prior transcriptome analysis results of robust immune and inflammatory responses triggered by PeV-A3 in airway epithelial cells (47). On the other hand, type I IFN treatment can inhibit PeV-A3 in neuronal cells, suggesting the PeV-A3 susceptibility of PeV-A3 to extracellular IFN treatment. The potential role of TLR in neuron injury by PeV-A3 infection was suggested (48); therefore, the expression of IFN genes and TLR-related pathway may act together to counteract PeV-A3 infection (49, 50).

Nevertheless, these endogenous antiviral responses seem unable to protect cells from PeV-A3-caused CPE. PeV-A3 might develop certain uncharacterized strategy to evade cellular IFN responses as other picornaviruses act (40, 51, 52). For example, a previous study showed that PeV-A1 induced interferon regulatory factor 3 (IRF3) phosphorylation and type I IFN production and STAT1/2 activation in airway cell type but not in intestinal cells (29, 53). Thus, the interplay between PeV-A3 and host antiviral activity merits further investigation.

The present results showed that PeV-A3 caused CPE in cells and induced death signaling activation and inflammatory responses *in vitro* and *in vivo*. Autophagy and pyroptosis are two types of programming cell death pathways accompanied with inflammatory responses (41, 54) that could be induced by PeV-A3 infection. This finding is also consistent with findings on other picornaviruses, such as Aichi virus, foot-and-mouth disease virus (FMDV), rhinovirus, enterovirus 71 (EV71), or coxsackievirus B3. Of note is that the infection-mediated autophagy and pyroptosis activation play different roles in promoting/limiting viral replication or antiviral interferon response regulation (40, 55–61). Thus, the effect of apoptosis, autophagy, and pyroptosis on PeV-A3 replication or antiviral immune regulation should be further explored in the future.

PeV-A3 has emerged as a neuroinvasive virus. Clinical evidences revealed that PeV-A3 induced innate immune responses of cytokine/chemokine expression in neonates and infants (62). However, there was no suitable animal model for understanding neonatal PeV-A3 (26). This study put forward the first neonatal infection animal model established for exploring PeV-A3 neuropathogenesis. In our model, we found the PeV-A3 infection in the hippocampus and cortex regions of the neonatal mouse brain. Results obtained using the model supported the clinical finding of innate inflammation response. Moreover, mechanistic analysis results revealed different cell death signaling pathways triggered by PeV-A3 in mice. This established PeV-A3 infection model would be useful for understanding the viral disease mechanism, which would in turn facilitate the development of effective prevention and treatment methods.

In addition to the PeV-A3 infection animal model in this study, other picornavirus infection animal models were previously established in mice to address critical issues. For example, the pathogenic mechanism of echovirus was revealed in the transgenic mice with human integrin very late antigen 2 (VLA-2, echovirus entry receptor) expression. In an intracerebral inoculation of echovirus in VLA-2 newborn mice, the mice showed paralysis and wasting (63). Compared with the echovirus infection animal model, although PeV-A3 entry receptor was not identified, we found that PeV-A3 could directly infect human cells and C57BL/6 mouse brain, suggesting that PeV-A3 infection may use a similar existing entry mechanism in different mammals. However, this hypothesis needs to be confirmed with further studies. EV71 is another neuropathic picornavirus. Two EV71 disease mouse models have been established before. One model used mouse-adaptive EV71 to infect 1-day-old Institute of Cancer Research (ICR) mice (64); the other used non-mouse adaptive EV71 to infect 3-day-old to 2-week-old AG129 mice (deficient in interferon alpha/beta and gamma receptor signaling) (65). Both studies revealed 100% mortality for intraperitoneal infection with EV71. However, this present study observed only 20% mortality in PeV-A3-infected newborn C57BL/6 mice. Such discrepancy may be attributed to viral virulence and mouse strain used. Contrary to using mouse-adapted EV71 strain or type I IFN activity-deficient mice, this study employed clinically isolated PeV-A3 to challenge immunocompetent mice, with induction of host IFN activity providing certain protection activity. In addition, oral infection of EV71 causing the disease in mice was also demonstrated (64, 65).

Although PeV-A3 infection was presumed to be fecal–oral and respiratory routes (9), direct evidence supporting PeV-A3 transmission *via* fecal-oral route is lacking (66). Thus, this study adapted the previously reported echovirus infection method (63), with PeV-A3 intracerebrally inoculated into newborn mice. Intrafamilial transmission of PeV-A and enteroviruses in neonates and young infants seems to be critical for spreading virus; hence, using different methods of viral inoculation to mimic PeV-A3 infections for further study would be of interest (67).

This study showed that type I IFN treatment was able to activate host innate immunity against PeV-A3 *in vitro*. Although this treatment was not administered in this study, similar results were expected for EV71, whose infection and replication in mice, though rare, were potently controlled by type I IFN (68). Except for the biologics treatment, the *in vitro*-identified small compounds or drugs against picornavirus need to be evaluated *in vivo* (69). The proposed PeV-A3 animal model would serve as a useful platform to evaluate the efficacy of antiviral compounds.

## Conclusion

This study successfully deciphered neuropathogenic mechanisms of PeV-A3 in human neuronal cells and in neonatal mice. PeV-A3-induced neuronal cell death-associated inflammation may play important roles in disease onset. The present findings enable better understanding of the characteristics and fundamental infection features of PeV-A3 in the neuronal system, which would contribute to the development of prevention or treatment method of PeV-A3 infection.

## Data Availability Statement

The original contributions presented in the study are included in the article/**Supplementary Material**. Further inquiries can be directed to the corresponding authors.

## Ethics Statement

The animal study was reviewed and approved by the Institutional Animal Care and Use Committee or Panel (IACUC) in Kaohsiung Veterans General Hospital, Taiwan (IACUC Approval No. VGHKS-2018-2021-A016).

## Author Contributions

Conceptualization: T-HC. Methodology: M-WJ, H-LS, T-HC, and KJT. Validation: T-HC and KJT. Formal analysis: M-WJ. Investigation: M-WJ, T-HC, and H-LS. Data curation: M-WJ, T-HC, and K-T. Writing—original draft preparation: M-WJ and T-HC. Writing—review and editing: H-LS, T-HC, and KJT. Supervision: T-HC and K-T. Funding acquisition: T-HC. All authors contributed to the article and approved the submitted version.

## Funding

This research was funded by the Ministry of Science and Technology of Taiwan, grant numbers MOST109-2320-B-016-013 and MOST110-2320-B-016-012, and by Kaohsiung Veterans General Hospital, grant numbers VGHKS107-170 and VGHKS 107-G02. The funders had no role in study design, data collection and analysis, decision to publish, or preparation of the manuscript.

## Conflict of Interest

The authors declare that the research was conducted in the absence of any commercial or financial relationships that could be construed as a potential conflict of interest.

## Publisher’s Note

All claims expressed in this article are solely those of the authors and do not necessarily represent those of their affiliated organizations, or those of the publisher, the editors and the reviewers. Any product that may be evaluated in this article, or claim that may be made by its manufacturer, is not guaranteed or endorsed by the publisher.

## Supplementary Material

Supplementary Figure 1 The panel of RT2 array.

Supplementary Figure 2 Cell death signaling analysis in PeV-A3 infected IMR-32 cells.
