# Supplementary material for: Characterization of Pathogenesis and Inflammatory Responses to Experimental Parechovirus Encephalitis
Source: Front Immunol. 2021 Nov 25;12:753683. doi: 10.3389/fimmu.2021.753683 (PMC8654935; doi:10.3389/fimmu.2021.753683)
Supplement: Supplementary Figure 1 — The panel of RT2 array. [file DataSheet_1.pdf]

Supplementary information

Table S1. The q-PCR primers used in this study.

Organism	Gene	Sequences (5’ - 3’)
Human	ATP6V1G2	F: AAC CCT CAC TGG GAC TTT GTA<br>R: CAA GAG AGG GAC CCA CAT CG
Human	BCL2A1	F: CAA GAG AGG GAC CCA CAT CG<br>R: TAC AAA GCC ATT TTC CCA GCC
Human	CD40	F: ACT GAT GTT GTC TGT GGT CCC<br>R: TGG CCA CCT TTT TGA TAA AGA CC
Human	DEFB1	F: AGA TGG CCT CAG GTG GTA AC<br>R: TCG GGC AGG CAG AAT AGA GA
Human	ESR1	F: ACC AGA TGG TCA GTG CCT TG<br>R: TGG ACC TGA TCA TGG AGG G
Human	FASLG	F: AAG AGA GGG AAC CAC AGC AC<br>R: GTG TGC ATC TGG CTG GTA GA
Human	FOXI1	F: CGG GCA AAG GGA ATT ACT GGA<br>R: TGA GGC TCC ATC CAA GAT GTC
Human	GAPDH	F: TGC ACC ACC AAC TGC TTA GC<br>R: GGC ATG GAC TGT GGT CAT
Human	HPeV3 VP1	F: AGG TGC CCC AAT TTC TTT TTC<br>R: AGC AGC CCC TGA ACC AGT T
Human	IFN1α	F: CCT CGC CCT TTG CTT TACT G<br>R: CAG AGA GCA GCT TGA CTT GCA
Human	IFNλ1	F: AGG CCC TGT CCC CAC TTC<br>R: GAG ATT TGA ACC TGC CAA TGT G
Human	IFNλ2/3	F: AGT TCC GGG CCT GTA TCC AG<br>R: GAG CCG GTA CAG CCA ATG GT
Human	IGF1	F: ATG TAT TGC GCA CCC CTC AA<br>R: GCA CTC CCT CTA CTT GCG TT
Human	IRGM	F: CGC TGA GGA CAT GGG AAA GA<br>R: AGT ATT CAC ATA CCC GCT CCT
Human	MAG	F: GCA CTG CCT CTG TTC TGG AT<br>R: TAC CAG ACA CCA TGC ACC AC
Human	MDA5	F: TGC TTC TCT AAG TGG GCA GC<br>R: TTT TCA CCC TGG CCC TGA AG
Human	OAS2	F: AAC TGC TTC CGA CAA TCA AC<br>R: CCT CCT TCT CCC TCC AAA A
Human	IL-6	F: TGT CCT GCA GCC ACT GGT TC

		R: AAG CCA GAG CTG TGC AGA TGA GTA
Human	TNFSR11B	F: AAG TGG ACC ACC CAG GAA AC<br>R: TGT GCC AGC TGT CTG TGT AG
Murine	HPRT	F: GCT CGA GAT GTC ATG AAG GAG AT<br>R: AAA GAA CTT ATA GCC CCC CTT GA
Murine	TNF	F: CAG CCG ATG GGT TGT ACC TT<br>R: GGC AGC CTT GTC CCT TGA
Murine	IFNα	F: TCC CCT CTC TCT TGC CTG AA<br>R: GCT GGG CAT CCA CCT TCT C
Murine	IFNβ	F: TGG CTA GGC CCT TTG CTT T<br>R: CTA GAG AGC AGG TTG ACC AGT AGC T
Murine	IFNγ	F: TCC TGC ACC AAC ATT TCT GA<br>R: TAC GAG GAC GGA GAG CTG TT
Murine	IRF3	F: CAC CCC AAG AAA ATC CAC TGA<br>R: AGG CGG TCA CCT CGA ACT C
Murine	IRF7	F: GAA GAC CCT GAT CCT GGT GA<br>R: CCA GGT CCA TGA GGA AGT GT
Murine	IL-1β	F: TTG ACG GAC CCC AAA AGA TG<br>R: TGG ACA GCC CAG GTC AAA G
Murine	IL-6	F: CCA CGG CCT TCC CTA CTT C<br>R: TTG GGA GTG GTA TCC TCT GTG A
Murine	TLR3	F: CCC AAT GGA AGA ACA AGA CCA A<br>R: AAG GAC GCC TGC TTC AAA GTC
Murine	Viperin	F: GGC GTG GAA GAA GCA ATA AAG T<br>R: CGC CAC GCT TCA GAA ACA

Figure S1

A

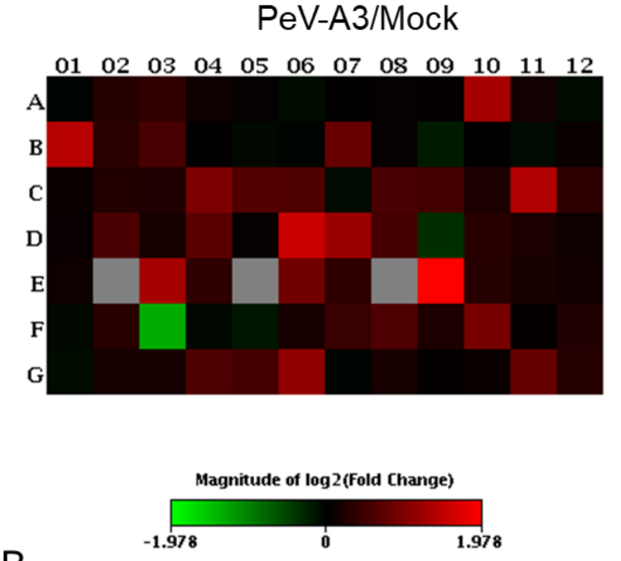

B

Position	Gene Symbol	Fold induction of PeV-A3/Mock
E09	MAG	3.94
D06	FASLG	2.96
B01	BCL2A1	2.69
C11	DEFB1	2.61
A10	ATP6V1G2	2.46
E03	IGF1	2.44
D07	FOXI1	2.30
G06	TNFSR11B	2.20

Figure S1. RT2 profiler PCR array in PeV-A3 infected GBM cells. (A) RT2 profiler PCR array was conducted in GBM cells infected with PeV-A3 or mock controls. Fold changes of the gene expression in infected cells and mock controls were calculated and colored as indicated. (B) Table of PeV-A3 induced genes with 2-fold expression (PeV-A3/ mock).

Table S2. Results of human cell death RT^2 profiler PCR array table on GBM cells.

Layout	01	02	03	04	05	06	07	08	09	10	11	12
A	ABL1<br>-1.02	AKT1<br>1.23	APAF1<br>1.29	APP<br>1.08	ATG12<br>1.03	ATG16L1<br>-1.07	ATG3<br>1.01	ATG5<br>1.04	ATG7<br>1.03	ATP6V1G2<br>2.46<br>A	BAX<br>1.12	BCL2<br>-1.07
B	BCL2A1<br>2.69<br>A	BCL2L1<br>1.26	BCL2L11<br>1.47	BECN1<br>1.01	BIRC2<br>-1.05	BIRC3<br>-1.03	BMF<br>1.73	C1orf159<br>1.04	CASP1<br>-1.15	CASP2<br>1.01	CASP3<br>-1.06	CASP6<br>1.05
C	CASP7<br>1.05	CASP9<br>1.20	CCDC103<br>1.19	CD40<br>1.94	CD40LG<br>1.53	CFLAR<br>1.52	COMMD4<br>-1.05	CTSB<br>1.47	CTSS<br>1.42	CYLD<br>1.17	DEFB1<br>2.61	DENND4A<br>1.28
D	DFFA<br>1.04	DPYSL4<br>1.49	EIF5B<br>1.14	ESR1<br>1.62	FAS<br>1.03	FASLG<br>2.96	FOXI1<br>2.30	GAA<br>1.44	GADD45A<br>-1.29	GALNT5<br>1.23	GRB2<br>1.15	HSPBAP1<br>1.07
E	HTT<br>1.08	IFNG<br>-1.08<br>C	IGF1<br>2.44	IGF1R<br>1.28	INS<br>-1.08<br>C	IRGM<br>1.83	JPH3<br>1.28	KCNIP1<br>-1.08<br>C	MAG<br>3.94	MAP1LC3A<br>1.22	MAPK8<br>1.13	MCL1<br>1.10
F	NFKB1<br>-1.04	NOL3<br>1.24	OR10J3<br>-2.53	PARP1<br>-1.05	PARP2<br>-1.13	PIK3C3<br>1.13	PVR<br>1.35	RAB25<br>1.52	RPS6KB1<br>1.16	S100A7A<br>1.89	SNCA<br>1.02	SPATA2<br>1.19
G	SQSTM1<br>-1.07	SYCP2<br>1.12	TMEM57<br>1.13	TNF<br>1.51	TNFRSF10A<br>1.43	TNFRSF11B<br>2.20	TNFRSF1A<br>-1.02	TP53<br>1.13	TRAF2<br>1.03	TXNL4B<br>1.06	ULK1<br>1.72	XIAP<br>1.22

Table S3. RT^2 and RT-qPCR analysis.

Gene symbol	Gene name	RT^2 array _fold change of PeV-A3/Mock	RT-qPCR _fold change of PeV-A3/Mock	p value (qPCR)	Description
MAG	Myelin-associated glycoprotein	3.94	2.24	<0.001	Type I membrane protein and member of the immunoglobulin superfamily. It is thought to be involved in the process of myelination. Myelin-associated glycoprotein is a major inhibitor of axonal regeneration in the CNS.
FASLG	Fas ligand (TNF superfamily, member 6)	2.96	2.46	<0.01	A member of the tumor necrosis factor superfamily. The function of the encoded transmembrane protein is the induction of apoptosis triggered by binding to FAS. The FAS/FASLG signaling pathway is essential for immune system regulation, including activation-induced cell death (AICD) of T cells and cytotoxic T lymphocyte-induced cell death.
BCL2A1	BCL2-related protein A1	2.69	2.46	<0.05	This gene is a direct transcription target of NF-kappa B in response to inflammatory mediators, and is up-regulated by different extracellular signals.
DEFB1	Defensin, beta 1	2.61	1.89	<0.01	Defensins form a family of microbicidal and cytotoxic peptides made by neutrophils.
ATP6V1G2	ATPase, H+ transporting, lysosomal 13kDa, V1 subunit G2	2.46	1.12	ND	Required for TNF-induced necrosis.
IGF1	Insulin-like growth factor 1 (somatomedin C)	2.44	1.87	<0.05	This gene is similar to insulin in function and structure and is a member of a family of proteins involved in mediating growth and development. IGF-1 receptor antagonism inhibits autophagy.

FOXI1	Forkhead box I1	2.3	1.71	<0.001	This gene plays an important role in the development of the cochlea and vestibulum, as well as embryogenesis. Foxl1-/- livers exhibit more extensive parenchymal necrosis than controls following bile duct ligation.
TNFRSF11B	Tumor necrosis factor receptor superfamily, member 11b	2.2	9.46	<0.001	This gene is a member of the TNF-receptor superfamily. Osteoprotegerin has two known TNF family ligands: receptor activator of NF-κB.

ND: non-determined.

Figure S2

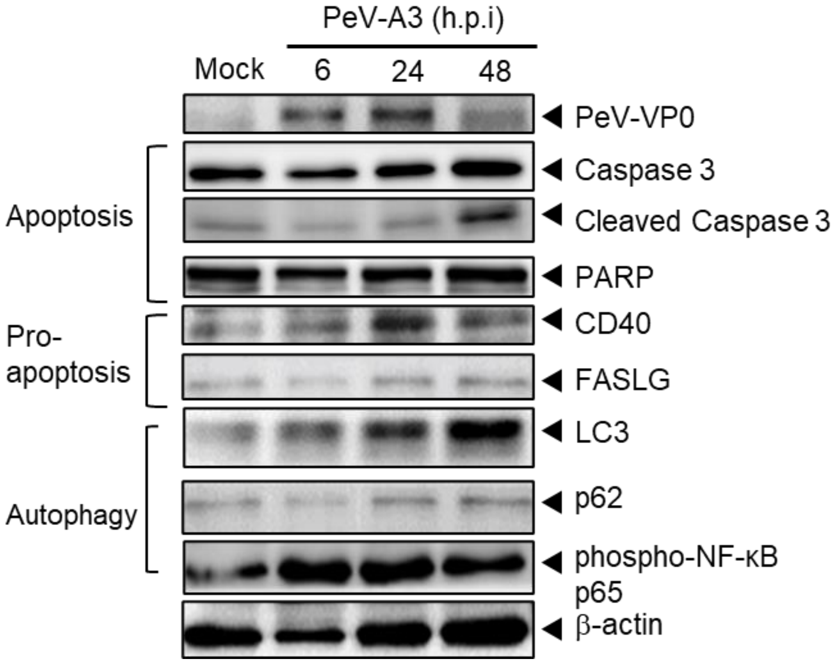

Figure S2. Cell death signaling analysis in PeV-A3 infected IMR-32 cells. **(A)** IMR-32 cells were infected with PeV-A3 at MOI = 1 for 6 h, 24 h and 48 h. Cell extracts were employed to analyze cell death signaling pathways by Western blotting using anti-PeV VP0, anti-caspase 3, anti-PARP, anti- anti-CD40, anti-FASLG, anti-LC3, anti-p62, anti-phospho-NF-κB p65 (Ser536) and anti-β-actin.
